# Rare Variants in the *TREX1* Gene and Susceptibility to Autoimmune Diseases

**DOI:** 10.1155/2013/471703

**Published:** 2013-10-09

**Authors:** Nadia Barizzone, Sara Monti, Simona Mellone, Michela Godi, Maurizio Marchini, Raffaella Scorza, Maria G. Danieli, Sandra D'Alfonso

**Affiliations:** ^1^Department of Health Sciences, University of Eastern Piedmont, 28100 Novara, Italy; ^2^Interdisciplinary Research Center of Autoimmune Diseases (IRCAD), University of Eastern Piedmont, 28100 Novara, Italy; ^3^Unit of Clinical Immunology and Allergology, Fondazione IRCCS Ca' Granda Ospedale Maggiore Policlinico and University of Milano, 20122 Milano, Italy; ^4^Sezione di Clinica Medica, Università Politecnica delle Marche & Ospedali Riuniti, 60121 Ancona, Italy

## Abstract

TREX1 (DNase III) is an exonuclease involved in response to oxidative stress and apoptosis. Heterozygous mutations in *TREX1* were previously observed in patients with systemic lupus erythematosus (SLE) and Sjögren's syndrome (SS). We performed a mutational analysis of the *TREX1* gene on three autoimmune diseases: SLE (210 patients) and SS (58 patients), to confirm a *TREX1* involvement in the Italian population, and systemic sclerosis (SSc, 150 patients) because it shares similarities with SLE (presence of antinuclear antibodies and connective tissue damage). We observed 7 variations; two of these are novel nonsynonymous variants (p.Glu198Lys and p.Met232Val). They were detected in one SS and in one SSc patient, respectively, and in none of the 200 healthy controls typed in this study and of the 1712 published controls. *In silico* analysis predicts a possibly damaging role on protein function for both variants. The other 5 variations are synonymous and only one of them is novel (p.Pro48Pro). This study contributes to the demonstration that *TREX1* is involved in autoimmune diseases and proposes that the spectrum of involved autoimmune diseases can be broader and includes SSc. We do not confirm a role of *TREX1* variants in SLE.

## 1. Introduction

TREX1 (DNase III) is a 3′–5′ exonuclease involved in the response to oxidative stress and apoptosis. It is active as a homodimer and normally associates to the endoplasmic reticulum, as part of a protein complex (SET complex) that is translocated to the nucleus in response to granzyme A-mediated cell death [1]. Moreover, Christmann et al. [2] have recently demonstrated that the TREX1 protein is upregulated and translocated to the nucleus in the cells treated with UV-C light (320 nm) and that murine trex1^−/−^ cells are more susceptible to DNA damage caused by UV. Trex1 knockout mice develop an inflammatory myocarditis which results in progressive cardiomyopathy, circulatory failure, and reduced survival [3].

Heterozygous mutations in the *TREX1* gene were observed in 9/417 patients with systemic lupus erythematosus (SLE) and in 1/169 subjects with Sjögren's syndrome (SS) [4]. Subsequently, de Vries et al. found an additional heterozygous missense variant in one out of 60 patients affected by neuropsychiatric-SLE (NPSLE) [5]. These variations were not found in 1712 controls [4]. 

Heterozygous mutations of *TREX1* have also been described in familial chilblain lupus (FCL), a rare autosomal-dominant form of cutaneous lupus erythematosus manifesting in early childhood [6]; in retinal vasculopathy with cerebral leukodystrophy (RVCL), an adult-onset disease characterized by central nervous system degeneration, retinal vasculopathy, and nephropathy [7]; and in a Taiwanese family with a hereditary small vessel disease of the brain clinically distinct from RVCL (CADASIL, cerebral autosomal dominant arteriopathy with subcortical infarcts and leukoencephalopathy) [8]. Furthermore, *TREX1 *mutations cause Aicardi-Goutières syndrome (AGS), a genetically heterogeneous, autosomal recessive disorder presenting with early-onset progressive encephalopathy [9]. It has been reported that some AGS patients show a partial clinical overlap with lupus erythematosus. Particularly, AGS patients may present lupus-like rash, oral ulcers, arthritis, thrombocytopenia, leukocytopenia, and presence of antinuclear antibodies (ANA) [9].

Altogether, these data highlight a relevant role of *TREX1* in autoimmune rheumatological diseases. On these bases, we decided to investigate the role of *TREX1* gene in susceptibility to three different autoimmune diseases in the Italian population: SLE, SS, and systemic sclerosis (SSc). In particular, this work aims to replicate in the Italian population the involvement of *TREX1* in SLE and SS, and to extend the investigation to a further autoimmune disease. SSc was included in the study because it shares some features with SLE, namely, the fact that they are both characterized by connective tissue inflammation and the presence of antinuclear autoantibodies. 

## 2. Materials and Methods

### 2.1. Subjects

We performed a mutational screening on 210 SLE, 58 SS, and 150 SSc patients. Furthermore, we analyzed 200 healthy control subjects, comprising blood donors. The geographic origin of controls and of patients of the three sample sets was similar. All subjects belonged to the Italian population and were unrelated. Individuals with Sardinian ancestors were excluded. All the samples were collected after informed consent and appropriate ethical approvals.

SLE patients (female : male ratio = 7.5 : 1) fulfilled at least 4 of the American College of Rheumatology 1997 revised criteria for the classification of SLE [10]. 

SS patients fulfilled the revised criteria proposed by the American-European Consensus Group [11].

SSc patients (female : male ratio = 13 : 1) fulfilled the American College of Rheumatology preliminary criteria for the classification of SSc [12]. Sixteen percent of the patients were affected by the diffuse cutaneous (dcSSc) and 84% by the limited cutaneous (lcSSc) form of the disease. Pulmonary hypertension, estimated by echocardiography, was defined as a right-ventricular systolic pressure ≥40 mm Hg and was observed in 29% of patients. Pulmonary fibrosis was evaluated by high resolution CT (HR-CT) and was present in 36% of SSc subjects. Disease onset was determined by the patient's recall of the first non-Raynaud symptom clearly ascribable to scleroderma. 

### 2.2. Search for Variations in *TREX1* Gene

Genomic DNA was isolated from peripheral blood with standard methods. The entire coding sequence of the *TREX1* gene (945 bp, NM_033629) is contained in one single exon. It was amplified in three overlapping fragments and analysed by Denaturing High Performance Liquid Chromatography (DHPLC) or direct sequencing. DHPLC temperatures of analysis were calculated with the algorithm “DHPLC melt program” (http://insertion.stanford.edu/melt), and the samples were processed with the Transgenomic WAVE (Transgenomic, Omaha, NE, USA). Results were analysed with the NavigatorTM software (Transgenomic, Omaha, NE, USA). Sequencing reactions were performed using the ABI PRISM BigDye Terminator kit v.1.1 (Applied Biosystems, Foster City, CA, USA) and analysed with the 3100 Genetic Analyser automatic sequencer (Applied Biosystems, Foster City, CA, USA). Primer sequences and analysis conditions are available on demand. The position of the detected variations is referred to the sequence NM_033629, (ATG = 1) from the NCBI public database. The detected missense variations were genotyped on 200 controls by DHPLC.

### 2.3. *In Silico* Analysis

The putative functional relevance of the detected missense variations was evaluated with the programs: PolyPhen (http://genetics.bwh.harvard.edu/pph), SIFT (http://sift.bii.a-star.edu.sg/), SNAP (https://rostlab.org/services/snap/), pMUT (http://mmb.pcb.ub.es/PMut/) and MutPred (http://mutpred.mutdb.org/). The putative effect of the variations on splicing sites was evaluated using the SpliceView program (http://www.itb.cnr.it/sun/webgene/) and the ESEfinder scoring matrix (http://rulai.cshl.edu/cgi-bin/tools/ESE3/esefinder.cgi?process=home). To analyse the impact of synonymous variants on gene and epigenetic regulation, we used ENCODE data available through the UCSC web server (http://genome-euro.ucsc.edu/) and confirmed them with the software ChroMoS (Chromatin Modified SNPs) (http://epicenter.immunbio.mpg.de/services/chromos/), which combines genetic and epigenetic data. Differential analysis of transcription factor binding was performed with sTRAP software (http://epicenter.immunbio.mpg.de/cgi-bin/chromos/sTrap.cgi).

## 3. Results

We detected 6 rare single nucleotide variants in the *TREX1 *coding sequence in 2/210 SLE, 3/58 SS, and 1/150 SSc patients (Table 1), plus a common SNP (p.Tyr177Tyr, rs11797). Two of the rare variants were nonsynonymous: c.592G>A (p.Glu198Lys) and c.694A>G (p.Met232Val) and were observed in one SS and one SSc heterozygous patient, respectively. The SSc patient was a female affected by the limited form of the disease with an age of onset of 49 years, who showed pulmonary hypertension and anticentromere autoantibodies. The p.Glu198Lys variant has recently been reported in homozygosity in one AGS patient [9], while p.Met232Val is novel. However, in an AGS patient, a Met232Hisfs∗9 caused by the insertion of a single base has been observed (LOVD—Leiden Open Variation Database), and a different missense substitution affecting the same residue (p.Met232Arg) has been reported in the Exome Variant Server (http://evs.gs.washington.edu/EVS/), observed in 1 out of 4406 individuals with African descent but in none out of 8600 individuals with European descent.

**Figure 1:**
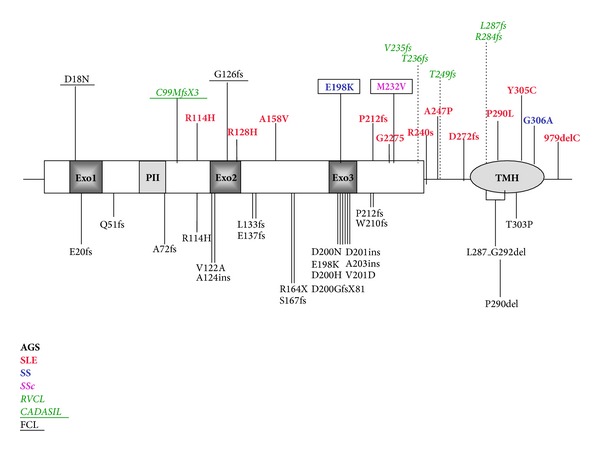
Scheme of disease-associated *TREX1* mutations so far reported in literature. The two missense mutations found in the present study are in squares. Normal type, black: AGS; *italic, green: RVCL*; *underlined, italic, green: CADASIL*; underlined black: FCL; boldface, red: SLE; boldface, pink: SSc; boldface, blue: SS. Exo 1, 2, 3 domains; PII: polyproline II domain; TMH: transmembrane domain.

An *in silico* analysis was performed to predict the possible functional role of the two missense variations, using five different prediction algorithms (PolyPhen, SIFT, SNAP, pMUT and MutPred). According to all four softwares, both substitutions have a possibly damaging effect. The p.Glu198Lys variant is located in the highly conserved third exonuclease (Exo3) domain, one of the three regions that, together with Exo1 and Exo2, form the catalytic core of the protein. Conversely, p.Met232Val falls in a region of uncertain function between the domain Exo3 and the membrane binding region TMH (Figure 1).

The two missense variants were not detected in 200 Italian healthy controls, nor were observed in further 1712 published controls [4] or in 8600 European American controls from the Exome Variant Server.

The other 5 detected variations were synonymous (Table 1). Only c.144 C>G (p.Pro48Pro) is novel, while the others have already been described in healthy controls [4] and in public databases. The c.531C>T - (p.Tyr177Tyr) variant is a common polymorphism of the *TREX1* gene (rs11797) and was observed with an allelic frequency of 0.42–0.47 in the three patient sample sets, with a similar frequency to that reported in the literature (0.42) [4] and in public databases (0.38, NCBI). Conversely, the other four (Table 1) are rare variants in our dataset and in literature.

According to the Spliceview algorithm none of the variants affects canonical splice sites. Conversely, the analysis performed with ESEfinder showed that c.144 C>G, c.592 G>A, and c.912 G>A might have some functional effect by introducing or removing a site of interaction with splicing proteins. Furthermore, the analysis of the relative codon usage frequencies among synonymous codons showed that both c.144 C>G and c.912 G>A cause the introduction of a rarer codon (c.144 C>G: 0.11 versus 0.33; c.912 G>A: 0.07 versus 0.43). According to ENCODE and ChroMoS, based on chromatin status, all SNPs are located in a very active region which may have strong promoter activity. However, the subsequent analysis with sTRAP, aimed at identifying differential transcription factor binding, shows only weak differences in the binding affinities; the greatest difference, expressed as log(*P* value), was observed for c.198 G>A (G *P* = 0.818; A *P* = 0.040, difference log⁡(*P*) = 1.306). Nominal significant differences were reported also for c.462 T>C and c.912 G>A. 

## 4. Discussion 

This work stems from recent papers which strongly suggest a role of *TREX1* in autoimmune diseases. In this study, we analysed patients affected by three different autoimmune diseases, namely, SLE, SS, and SSc belonging to the Italian population. 

We identified two *TREX1* missense mutations, each in one patient, which were not observed in the 200 Italian controls analysed in this study nor in 1712 controls from the literature, fully sequenced for the *TREX1* coding region by Lee-Kirsch et al. [4].

One of them is a novel *TREX1* mutation (p.Met232Val) identified in an SSc patient affected by the limited form of the disease. This substitution affects a conserved nucleotide, and *in silico* analysis performed with five prediction algorithms predicts for it a nonneutral role. MutPred software predicts a possible effect on protein folding (gain of sheet). This position is not located in a functional region but falls in the protein portion comprised between the exonucleasic domain and the membrane binding domain TMH. Several mutations, both frameshift and nonsynonymous, located in the same protein portion, have been reported in SLE, AGS, and in RVCL patients (Figure 1). Although most of them were frameshift variants, missense mutations in this region (p.Gly227Ser, p.Arg240Ser, and p.Ala247Pro) were described in two Afrocaribbean patients with SLE and secondary SS (p.Gly227Ser and p.Ala247Pro were found on the same allele) [4]. These variants were not functionally characterized. Therefore, functional analysis is needed to clarify the possible functional relevance of p.Met232Val. The second mutation (p.Glu198Lys) was observed in an SS patient. This variant was previously reported in homozygosity in a 19-year-old Turkish AGS patient with lupus-like features: chilblain lesions on feet, oral ulcers, ANA (antinuclear antigens), anti-ENA (extractable nuclear antigens), and decreased C3 levels [9]. Clinical information about the parents was not available; thus, the phenotype of heterozygous individuals is not known. The substitution affects the functional domain Exo3, and it is predicted as damaging by *in silico* analysis. The importance of this sequence in the functionality of the enzyme is strongly suggested by the fact that several other mutations affecting the Exo3 domain have been reported in AGS patients, both in homozygous and in heterozygous form (Figure 1). In vitro functional analysis has been performed for some of these mutations (p.Asp200Asn, p.Asp201ins, and p.Val201Asp), demonstrating a dramatic decrease (from 4-fold for p.Val201Asp up to 35,000-fold for p.Asp201ins) in the exonuclease activity [13, 14]. Intriguingly, both mutations involving Asp200 (p.Asp200Asn and p.Asp200His, Figure 1) seem to be sufficient, in heterozygosity, to cause AGS, usually a recessive disease [9, 14]. A dominant negative effect can be hypothesized, since Lehtinen et al. [14] demonstrated with experiments of coexpression in *E. Coli* cells, protein purification and with an exonuclease assay that TREX1^200Asn^ inhibits DNA degradation even in the presence of TREX1^wt^. In fact, the TREX1^wt/200Asn^ heterodimer dsDNA activity showed a 200-fold decrease if compared to TREX1^wt^ dsDNA activity. Nuclease activity on ssDNA showed only a slight decrease (1.5-fold). The authors suggested that TREX1^200Asn^ acts with a dominant negative effect, inhibiting TREX1^wt^ DNA degradation.

Considering the physiological role of the TREX1 protein, it is tempting to speculate that a defective TREX1 may result in the failure to degrade ssDNA or dsDNA leading to immune activation and development of autoantibodies against these macromolecules. 

In addition to the two missense mutations, we observe also five synonymous variants. *In silico* analysis was performed to predict functional impact on splicing, epigenetic, and transcription factors binding, with only weak and inconclusive evidences, therefore, we can not consider these variations as functionally related with the disease.

The present study confirms the presence of *TREX1* mutations in patients affected by SS with a frequency of 1/58. This value is not statistically different from that reported in the literature (1/169). The overall allelic frequency of all *TREX1* rare variants (Minor Allele Frequency < 1%) in SS patients (4/58) is statistically higher than in the 4300 healthy European American individuals from the Exome Variant Server (Peto's OR = 27.2, 95% CI = 3.5–208.8, Fisher's exact test 2-tailed *P* value = 0.025). In order to restrict the statistical analysis only to the functional variations, we have functionally annotated the nonsynonymous variants of the Exome Variant Server with the same algorithms used for the *in silico* analysis on our mutations, observing one frameshift and 5 missense variants predicted as damaging by at least 3 softwares. One of these was the p.R114H, which is the most common AGS-causing variant in homozygosity or compound heterozygosity. The frequency of this mutation on the Exome Variant Server is consistent with the expected carrier frequency in the general population, based on AGS prevalence (<1/1 000 000) as reported by Orphanet. The frequency of potentially pathological variants is higher in our SS subset than in the Exome Variant Server healthy controls (Peto's OR = 208.3, 95% CI = 1.5–29137.8). The finding of a mutation, previously reported as deleterious, in an SS patient strengthens the hypothesis of a *TREX1* involvement in this disorder. 

Moreover, this study identifies for the first time a *TREX1* mutation in one SSc patient. This frequency (1/158) is higher than in the Exome Variant Server healthy controls (Peto's OR = 4.60), but the difference is not significant. The involvement of *TREX1* in this disease has been only poorly analyzed so far, limited to an association study on 4 common polymorphisms [15]. The authors did not observe any significant association, but this does not exclude the existence of rare variants involved with the disease. Our study, reporting the identification of one damaging *TREX1* mutation in SSc, suggests a possible involvement of *TREX1* in SSc, which may deserve a confirmation in further studies. Conversely, we found only 2 synonymous substitutions in our 210 SLE patients, thus, not replicating in the Italian population, the results observed in 417 patients from Northern Europe (UK, Germany and Netherlands), where 9 missense or frameshift mutations were detected (Figure 1) leading to a *TREX1* mutation frequency of 0.021. More recently, however, a large genetic scan over 15864 samples from Oklahoma was performed, but only known variants were analysed [16]. The observed overall frequency of nonsynonymous mutations was 0.005, lower than that reported by Lee-Kirsch et al. [4]. Our data is consistent with Namjou's analysis, in fact, given this new frequency, we would expect to observe zero or one mutation. 

## 5. Conclusions 

In conclusion, this study contributes to the demonstration that *TREX1* is involved in autoimmune diseases and proposes that the spectrum of involved autoimmune diseases can be broader, since we detected a previously unreported possible association between *TREX1* mutations and SSc. Moreover, our study confirms that rare private variants may play a role in the susceptibility to multifactorial diseases.

## Figures and Tables

**Table 1:** *TREX1* variants.

Nucleotide variation	Amino acid change	ID, reference	SLE *N* = 210	SSc *N* = 150	SS *N* = 58
c.592G>A	**p.Glu198Lys**	Ramantani et al. 2010 [9]	0	0	**1**
c.694A>G	**p.Met232Val**	Novel	0	**1**	0
c.144C>G	p.Pro48Pro	Novel	**1**	0	0
c198G>A*	p.Lys66Lys	rs3135943 Lee-Kirsch et al. 2007 [4]	0	0	**1**
c.462T>C*	p.Asp154Asp	rs3135944 Lee-Kirsch et al. 2007 [4]	0	0	**1**
c.531C>T	p.Tyr177Tyr	rs11797 Lee-Kirsch et al. 2007 [4]	110	75	**29**
c.912G>A	p.Leu304Leu	rs3135945 Lee-Kirsch et al. 2007 [4]	**1**	0	**1**

Nucleotide numbering of *TREX1* variations reflects cDNA numbering with +1 corresponding to the A of the ATG translation initiation codon in the GenBank reference sequence NM_022517.17.

Besides c.531C>T, all the other variations were found only in heterozygosity.

*The two variants were observed in the same SS patient.
